# Supplementary material for: Distribution and Molecular Diversity of Whitefly Species Colonizing Cassava in Kenya
Source: Insects. 2021 Sep 27;12(10):875. doi: 10.3390/insects12100875 (PMC8541676; doi:10.3390/insects12100875)
Supplement: Supplementary file 1 [file insects-12-00875-s001.zip › insects-1335591-supplementary.pdf]

Molecular diversity of whitefly species colonizing cassava in Kenya and application of KASP genotyping to identify *Bemisia tabaci* haplotypes

Florence M. Munguti[1, 2*], Dora C. Kilalo[1] , Everlyne N. Wosula[4], Isaac Macharia[2], Evans N. Nyaboga[3], Agnes W. Mwango'mbe[1]

[1]Department of Plant Science and Crop Protection, University Nairobi, P.O. Box 29053 - 00625, Nairobi, Kenya

[2]Kenya Plant Health Inspectorate Service, P.O. Box 49592 - 00100, Nairobi, Kenya

[3]Department of Biochemistry, University Nairobi, P.O. Box 30197 - 00100, Nairobi, Kenya

[4]International Institute of Tropical Agriculture, P.O. Box 34441 Dar es Salaam, Tanzania

***Correspondence: munguti.florence@gmail.com**

Supplementary Table S1: Whitefly samples, sampled sites and whitefly species detected in cassava in Kenya based on MtCO1 sequences

	Sample ID	Date of collection	Region	County	subcounty	Latitude	Longitude	Altitude	Whitefly species	Genbank Accession No.
1	T05	3rd March 2020	Coastal	Taita-Taveta	Taveta	S 3˚26'36	E 37˚ 39'29	728	*Bemisia tabaci*	MT511672.1
2	T09	4th March 2020	Coastal	Taita-Taveta	Taveta	S 3˚16'37	E 37˚ 43'52	901.8	*Bemisia tabaci*	MT511672.1
3	T12	5th March 2020	Coastal	Taita-Taveta	Taveta	S 3˚11'59	E 37˚ 42'37	997.5	*Bemisia tabaci*	MT511672.1
4	T14	6th March 2020	Coastal	Taita-Taveta	Taveta	S 3˚15'9	E 37˚ 44'47	920.1	*Bemisia tabaci*	MT511672.1
5	E51	19th March 2019	Coastal	Taita-Taveta	Voi	-3.62081	38.85532833	453.5	*Bemisia tabaci*	LR535717.1
6	E53	19th March 2019	Coastal	Taita-Taveta	Voi	-3.640938333	38.70600167	652.6	*Bemisia tabaci*	LR535717.1
7	E54	19th March 2019	Coastal	Taita-Taveta	Voi	-3.844183333	38.64755167	571	*Bemisia tabaci*	LR535717.1
8	T01	3rd March 2020	Coastal	Taita-Taveta	Taveta	S 3˚26'52	E 37˚ 39'37	744.2	*Bemisia tabaci*	MT872661.1
9	E1	12th March 2019	Coastal	Kilifi	Kaloleni	-3.8494	39.527305	179.3	*Bemisia tabaci*	MT880240.1
10	E04	12th March 2019	Coastal	Kilifi	Mariakani	-3.858155	39.546865	181.3	*Bemisia tabaci*	MT511672.1
11	E07	12th March 2019	Coastal	Kilifi	Kilifi south	-3.929208333	39.725125	25.3	*Bemisia tabaci*	MT511672.1
12	E8	13th March 2019	Coastal	Kilifi	Kibarani	-3.557296667	39.824015	84.1	*Bemisia tabaci*	MT511672.1
13	E16	14th March 2019	Coastal	Kilifi	Kilifi North	-3.396845	39.91928333	23.4	*Bemisia tabaci*	LR535717.1
14	E18	14th March 2019	Coastal	Kilifi	Malindi	-3.248023333	40.04303	28.9	*Bemisia tabaci*	LR535717.1
15	E20	14th March 2019	Coastal	Kilifi	Malindi	-3.247791667	40.04517167	34.1	*Bemisia tabaci*	LR535717.1
16	E22	14th March 2019	Coastal	Kilifi	Malindi	-3.295675	40.028695	34.6	*Bemisia tabaci*	LR535717.1
17	E38	17th March 2019	Coastal	Kwale	Msambweni	-4.479955	39.454435	4.3	*Bemisia tabaci*	LR535717.1

18	E39	17th March 2019	Coastal	Kwale	Msambweni	-4.487131667	39.44866	12.9	*Bemisia tabaci*	LR535717.1
19	F9	13th March 2019	Coastal	Kilifi	Kilifi south	-3.932971667	39.73796	25.9	*Bemisia tabaci*	MT880240.1
20	E30	16th March 2019	Coastal	Kwale	Matuga	-4.187961667	39.49278167	290.2	*Bemisia tabaci*	MT872661.1
21	E31	16th March 2019	Coastal	Kwale	Golini	-4.15615	39.456125	357.9	*Bemisia tabaci*	LR535717.1
22	E47	16th March 2019	Coastal	Kwale	Lungalunga	-4.469221667	39.29222333	65	*Bemisia tabaci*	MT880240.1
23	E50	19th March 2019	Coastal	Kwale	Kinango	-3.733211667	39.04596	384.4	*Bemisia tabaci*	LR535717.1
24	F32	16th June 2019	Coastal	Kwale	matuga	-4.201893333	39.398555	182.6	*Bemisia tabaci*	MT880240.1
25	AT	13th March 2019	Coastal	Kilifi	Kilifi south	-3.930771667	39.72531833	26.7	*Bemisia tabaci*	LR535717.1
26	E2	12th March 2019	Coastal	Kilifi	Kaloleni	-3.839446667	39.54049333	176.4	*Bemisia tabaci*	LR535717.1
27	E53	19th March 2019	coastal	Taita-Taveta	Voi - Maungu	-3.64093833	38.70600167	652.6	*B. tabaci* SSA2	MT872660.1
28	T03	3rd March 2020	Coastal region	Taita-Taveta	Taveta	S 3˚26'54	E 37˚ 38'41	726.8	*A. dispersus*	KC822647.1
29	T04	4th March 2020	Coastal region	Taita-Taveta	Taveta	S 3˚26'47	E 37˚ 39'9	737.8	*A. dispersus*	KR063274.1
30	T24	5th March 2020	Coastal region	Taita-Taveta	Kaloleni	S 3˚28 '15	E 38˚ 25'9	836.1	*A. dispersus*	KY574540.1
31	E7	6th March 2020	Coastal region	Kilifi	Kilifi south	-3.929208333	39.725125	25.3	*A. dispersus*	KC822647.1
32	E38-C	7th March 2020	Coastal region	Kwale	Msambweni	-4.479955	39.454435	4.3	*A. dispersus*	KC822647.1
33	E-3B-C	8th March 2020	Coastal region	Kilifi	Kaloleni	-3.88587	39.55497833	169.7	*A. dispersus*	KC822648.1
34	T02	3rd March 2020	Coastal	Taita-Taveta	Taveta	S 3˚26'33	E 37˚ 38'36	726.6	*P. bondari*	MW488198.1
35	T13	4th Mach 2020	Coastal	Taita-Taveta	Taveta	S 3˚14'55	E 37˚ 45'1	923	*P. bondari*	MW488198.1
36	T46	7th July 2018	Coastal	Kilifi, Kaloleni	Kaloleni	E039˚ 35.415	S0˚ 3 50.229	211	*P. bondari*	MW488198.1

37	T18	6th March 2020	Coastal	Taita-Taveta	Mwatate	S 3˚30'8	E 38˚ 17'47	941	*P. bondari*	MW041899.1
38	ME6	1st June 2020	Eastern	Makueni	Mbuvo	-1.963378333333333	37.76287333333333	1043.0	*Bemisia tabaci*	MT880240.1
39	ME18	2nd June 2020	Eastern	Kitui	Mingwani	-0.9710016666666667	37.896748333333335	974.9	*Bemisia tabaci*	MT880240.1
40	ME 19	3rd June 2020	Eastern	Kitui	Mingwani	-0.983381666666666	37.88912333333334	1090.9	*Bemisia tabaci*	: MT880240.1
41	ME23	4th June 2020	Eastern	Machakos	Masinga	-0.964461667	37.68635	1222.3	*Bemisia tabaci*	MT880240.1
42	ME 24	5th June 2020	Eastern	Embu	Mbeere	-0.6274200000000001	37.521085	1216.2	*Bemisia tabaci*	MT880240.1
43	ME 12	12th June 2020	Eastern	Makueni	Mbooni East	-1.679148333	37.58676333333334	1251.6	*B. afer*	KF734668.1
44	ME1	10th June 2020	Eastern	Machakos	Kalama, KALRO Katumani	-1.5848650000000002	37.23970833333333	1618.3	*B. afer*	KF734668.1
45	ME 13	12th June 2020	Eastern	Makueni	Mbooni East	-1.6755816666666665	37.57884833333333	1235.2	*B. afer*	AF418673.2
46	ME 10	11th June 2020	Eastern	Makueni	Makueni	-1.847118333		37.65136	1162.3	Mitochondrion Bemisia tabaci
47	ME25	6th June 2020	Central	Muranga	Kiharu	-0.69102	37.187763333333336	1227.5	*Bemisia tabaci*	MT880240.1

48	ME 27	14th June 2020	Central	Murang'a	Ikidu	-0.7681783333333333	37.1543066666667	1308.3	*B. afer*	KF734668.1
49	ME28	14th June 2020	Central	Murang'a	Mobp	-0.889265	37.12565166666666	1367.8	*B. afer*	KF734668.1
50	ME 26	4th June 2020	Central	Murang'a	Murang'a	-0.725281667	37.17183	1253.8	*Bemisia tabaci*	KY951448.1
51	MW9	29th June 2020	Western	Busia	Teso south	0.45738333333333336	34.164750000000005	1195.4	*Bemisia tabaci*	MT872662.1
52	MW10	30th June 2020	Western	Busia	Teso south	0.47551	34.19762166666667	1208.6	*Bemisia tabaci*	MT872661.1
53	MW6	29th June 2020	Western region	Busia	Teso south	0.588978333	34.215295	1095.7	*Bemisia tabaci*	MT872661.1
54	MW7	29th June 2020	Western region	Busia	Teso south	0.533526666	34.16453833	1147.8	*Bemisia tabaci*	
55	MW28	1st July 2020	Western region	Busia	Teso south	-1.18861	34.45367666	1499.8	*Bemisia tabaci*	MT872661.1
56	MW52	1st July 2020	Western region	Busia	Teso south	-0.73026	34.57834833	1417.1	*Bemisia tabaci*	MT872661.1
57	W6	7th June 2019	Western region	Kakamega	Kwhisero	0.16336	34.60133167	1468.2	*Bemisia tabaci*	MT872661.1
58	W12	5th June 2019	Western	Kakamega	Matungu	0.396136667	34.49103667	1295.7	*B. tabaci* SSA2	MT872660.1
59	W20	6th June 2019	Western	Bungoma	Bungoma central	0.753301667	34.65445	1554.9	*B. tabaci* SSA2	MK940754.1
60	W29	6th June 2019	Western	Bungoma	Kimilili	0.805953333	34.76315667	1670.7	*B. tabaci* SSA2	MT872660.1
61	W35	7th June 2019	Western	Teso North	Amagoro	0.622988333	34.34048833	1164.4	*B. tabaci* SSA2	MT872660.1
62	W44	8th June 2019	western	Busia	Teso south	0.593996667	34.222295	1150.9	*B. tabaci* SSA2	MT872660.1
63	MW4	29th June 2020	Western	Busia	Teso North	0.691241666	34.32152667	1322.6	*B. tabaci* SSA2	MT872660.1

64	MW44	3rd July 2020	Nyanza	Migori	Kuria West	-1.2315716666666667	34.504839999999994	1593.4	*Bemisia tabaci*	MT872662.1
65	MW12	30th June 2020	Nyanza	Kisumu	Kisumu west	-0.338473333	34.812733333333334	1178.9	*Bemisia tabaci*	MT872661.1
66	MW15	30th June 2020	Nyanza	Homabay	Homabay-Town	-0.6432533333333333	34.46548	1353	*Bemisia tabaci*	MT872661.1
67	MW30	1st July 2020	Nyanza	Migori	Kuria-west	-1.1146683333333334	34.5151716666667	1402.0	*Bemisia tabaci*	MT559300.1
68	MW42	3rd July 2020	Nyanza	Migori	Kuria-west	-1.188751667	34.4237716666667	1460.1	*Bemisia tabaci*	MT559300.1
69	MW11	30th June 2020	Nyanza	Kisumu	Kisumu west	-0.29252	34.94606833333333	1156.3	*Bemisia tabaci*	MT872661.1
70	MW13	30th June 2020	Nyanza region	Homabay		-0.338473333	34.812733333333334	1178.9	*Bemisia tabaci*	MT559300.1
71	MW 17	30th June 2020	Nyanza	Homabay	Dhiwa	-0.6412302	34.5449365	1363.7	*B. afer*	KF734668.1
72	MW41	3rd July 2020	Nyanza	Migori	Kuria West	-1.1701833333333334	34.440799999999996	1460.6	*B. afer*	KF734668
73	MW 26	1st July 2020	Nyanza	Migori	Uriri	-0.9770133333333334	34.502721666666666	1401.7	*B. afer*	KF734668.1
74	MW 18	30th June 2020	Nyanza	Homa bay	Rangwe	-0.5988066666666667	34.567715	1301.3	*B. afer*	KF734668.1
75	MW 43	3rd July 2020	Nyanza	Migori	Kuria-East	-1.190213333	34.401765	1458.3	*B. afer*	KF734668.1
76	MW20/W20	1st July 2020	Nyanza	Homabay	Rangwe	-0.581875	34.629675	1479.6	*B. tabaci* SSA2	MK940754.1

Supplementary Table S2: Identification of *Bemisia tabaci* haplotypes in selected whitefly samples based on mtCO1 gene sequencing and KASP genotyping

Serial No.	Region	Sample ID	Haplotypes based on mtCO1
1	Coastal	E30	SSA1-SG1
2	Coastal	T01	SSA1-SG1
3	Coastal	E37	-
4	Coastal	T20	SSA1-SG3
5	Coastal	T05	SSA1-SG3
6	Coastal	T45B	SSA1-SG3
7	Coastal	T14	SSA1-SG3
8	Coastal	E47A	SSA1-SG3
9	Coastal	T12	SSA1-SG3
10	Coastal	E54	SSA1-SG3
11	Coastal	T35	SSA1-SG3
12	Coastal	T31	SSA1-SG3
13	Coastal	E39	SSA1-SG3
14	Coastal	T9	SSA1-SG3
15	Coastal	E22	SSA1-SG3
16	Coastal	E5	SSA1-SG3
17	Coastal	T54A	SSA1-SG3
18	Eastern	ME25	SSA1-SG3
19	Eastern	ME6	SSA1-SG3
20	Eastern	ME3	SSA1-SG3

21	**Eastern**	ME23	SSA1-SG3
22	**Eastern**	ME24	SSA1-SG3
23	**Eastern**	ME19	SSA1-SG3
24	**Eastern**	ME18	SSA1-SG3
25	**Western**	MW9	SSA1-SG2
26	**Western**	MW10	SSA1-SG1
27	**Western**	MW6	SSA1-SG1
28	**Western**	MW7	SSA1-SG1
29	**Western**	MW28	SSA1-SG1
30	**Western**	MW52	SSA1-SG1
31	**Nyanza**	MW44	SSA1-SG2
32	**Nyanza**	MW36	SSA1-SG2
33	**Nyanza**	MW12	SSA1-SG1
34	**Nyanza**	MW15	SSA1-SG1
35	**Nyanza**	MW30	SSA1-SG1
36	**Nyanza**	MW42	SSA1-SG1
37	**Nyanza**	MW11	SSA1-SG1
38	**Nyanza**	MW13	SSA1-SG1
